# Understanding the Pathway of Gas Hydrate Formation with Porous Materials for Enhanced Gas Separation

**DOI:** 10.34133/2019/3206024

**Published:** 2019-05-28

**Authors:** Jia Liu, Yajuan Wei, Wei Meng, Pei-Zhou Li, Yanli Zhao, Ruqiang Zou

**Affiliations:** ^1^Division of Chemistry and Biological Chemistry, School of Physical and Mathematical Sciences, Nanyang Technological University, 21 Nanyang Link, Singapore 637371; ^2^Department of Chemistry, School of Science, Tianjin University, Tianjin 300072, China; ^3^School of Chemistry, Nankai University, Tianjin 300071, China; ^4^Department of Materials Science and Engineering, College of Engineering, Peking University, Beijing 100871, China

## Abstract

The reason that the stoichiometry of gas to water in artificial gas hydrates formed on porous materials is much higher than that in nature is still ambiguous. Fortunately, based on our experimental thermodynamic and kinetic study on the gas hydrate formation behavior with classic ordered mesoporous carbon CMK-3 and irregular porous activated carbon combined with density functional theory calculations, we discover a microscopic pathway of the gas hydrate formation on porous materials. Two interesting processes including (I) the replacement of water adsorbed on the carbon surface by gas and (II) further replacement of water in the pore by gas accompanied with the gas condensation in the pore and growth of gas hydrate crystals out of the pore were deduced. As a result, a great enhancement of the selectivity and regeneration for gas separation was achieved by controlling the gas hydrate formation behavior accurately.

## 1. Introduction

Gas hydrates are a type of ice-like clathrate compounds that existed in nature, which have attracted considerable attention because of their unique properties [1–5] and various applications. For example, artificial gas hydrates are considered one of the most promising materials for gas storage [6–8] and separation [9–11]. The formation of gas hydrates in hydrophobic pores has been widely reported as an effective method to enhance gas hydrate formation kinetics and capacity [12–18]. It has been reported that the stoichiometry of gas to water in artificial gas hydrates formed on porous carbon is much higher than that calculated from the crystal structure [9, 19]. An acceptable hypothesis is that the enhanced gas in artificial gas hydrates is attributed to additional gas adsorption in gaps between formed gas hydrates and pore surface or semiclathrate. However, important questions about what kind of gaps and how they can be formed are still not clarified, because there is no valid scientific method to distinguish this type of gaps and semiclathrate in the pore, and quantitative analysis of the gas in gas hydrates or adsorptive phase is hardly carried out. Some techniques such as molecular simulations [11, 20],* in situ* Raman spectroscopy [21], solid-state nuclear magnetic resonance [10, 22], neutron diffraction [23, 24], and synchrotron X-ray powder diffraction [25] could only be employed to confirm that the formation of gas hydrates is dependent on porous materials. There is no doubt that gas hydrates could be formed on porous materials, but the details about the formation process and whether they are formed inside the pores or not are still ambiguous.

**Figure 1:**
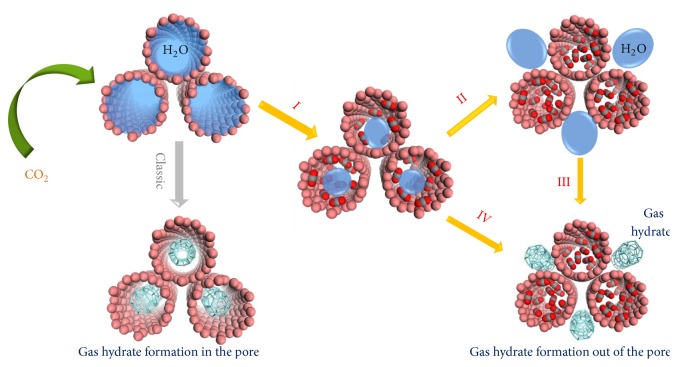
*Gas hydrate formation based on porous materials*. (I-IV) Replacing water by CO_2_ on the surface and in the pore, and large gas hydrate crystals are formed outside the pore.

A common understanding is that porous materials could be employed to enhance the gas hydrate formation due to the nanoconfinement effect. After the clusters of gas hydrates form in the nanoconfined space, blank space appears to allow for the gas occurrence as shown in Figure 1. Due to lack of convincing characterization techniques, however, the formation of gas hydrates in the confined space has not been experimentally proven yet. In comparison with this classic mechanism, we discovered a different process (I-IV in Figure 1) for the gas hydrate formation based on the investigation of CO_2_ hydrate formation on porous carbon. In this case, when CO_2_ was introduced into porous carbon fully filled with water, the considerable sorption of CO_2_ was observed on account of a consequence of interesting processes including (I) the replacement of water adsorbed on the carbon surface by CO_2_ and (II-IV) further replacement of water in the pore by CO_2_ accompanied with the growth of gas hydrate crystals out of the pore.

In process (I), although the calculated binding energy of C-CO_2_ is slightly weaker than that of C-H_2_O, a portion of water directly adsorbed on the carbon surface could still be replaced by CO_2_ driven by the entropy. During the process (II), the water inside the pore is further replaced by CO_2_ upon increasing CO_2_ chemical potential. Finally, gas hydrates are formed outside the confined space, and CO_2_ is condensed in the pore by route III. The gas hydrates could also be formed by following another route (IV). When the pressure reaches the value for the CO_2_ hydrate formation, microhydrate crystals initially form in the nanoconfined space and then move out to grow into large crystals in order to decrease the surface free energy. Meanwhile, the CO_2_ adsorption and condensation occur in the pore. Therefore, it was deduced that the gas hydrates could not be formed in the nanoconfined space. The excess gas observed in artificial gas hydrates resulted from the gas adsorption and condensation in the nanoconfined space when water is driven to form gas hydrates outside the pore. To confirm this assumption, we designed a rigorous experiment to investigate the gas hydrate formation behavior in the nanoconfined space and achieved a novel understanding about the formation behavior of gas hydrates on porous carbon materials. The pathway is totally different with previous hypothesis of the gas hydrate formation inside nanopores. Furthermore, a great enhancement of the selectivity and regeneration for the gas separation on porous materials was achieved based on the mechanism we proposed.

## 2. Results

**Figure 2:**
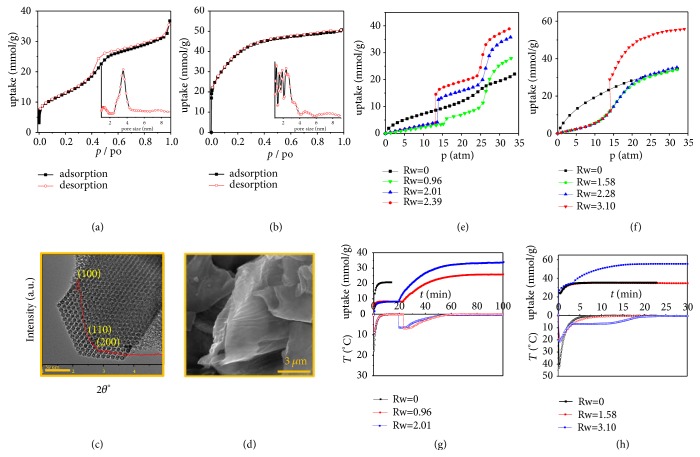
*Materials characterizations and experimental studies of gas hydrate formation process*. N_2_ adsorption/desorption isotherms on (a) CMK-3 and (b) AC at 77 K with DFT pore size distribution; (c) TEM image of CMK-3 (inset: powder XRD pattern) and (d) SEM image of AC; CO_2_ adsorption isotherms with different water ratios (*Rw*) at 273 K on (e) CMK-3 and (f) AC; CO_2_ dynamic sorption on (g) CMK-3 and (h) AC with different* Rw* at 273 K with an initial pressure of 30 atm. The filled points are corresponding to CO_2_ uptake; blank points are corresponding to temperature.

We selected CO_2_ as a representative gas for the study. The adsorptive CO_2_ could be condensed in confined space with a constant density easy for quantitative analysis. Thus, the CO_2_ hydrate formation on classic ordered mesoporous carbon CMK-3 and irregular porous activated carbon (AC) was investigated by thermodynamic experiments, thermodynamic calculations, kinetic analysis, density functional theory (DFT) calculation, and* in situ* Raman spectroscopy. The porous properties calculated from N_2_ isotherm curves at 77 K (Figures 2(a) and 2(b)) including Brumauer–Emmett–Teller (BET) surface area, DFT pore size, pore volume, and water vapor uptake (Figures [Supplementary-material supplementary-material-1] and [Supplementary-material supplementary-material-1]) are summarized in [Supplementary-material supplementary-material-1]. The pore volumes of CMK-3 and AC are 1.58 and 1.0 cm^3^/g, respectively. CMK-3 presents an ordered mesoporous structure as shown in Figure 2(c). The scanning electron microscopy (SEM) image of AC indicates a layered structure (Figure 2(d)).

When comparing CO_2_ isotherms determined on CMK-3 at 273 K with different mass ratios of water to carbon (*Rw*), obvious differences were observed as shown in Figure 2(e). The CO_2_ isotherm on dry CMK-3 presents a type IV isotherm classified by IUPAC, because of the condensation of CO_2_ in the mesopore (3.8 nm). When* Rw *= 1 (i.e., the pore is full of water), the CO_2_ uptake is obviously lower than that on the dry sample under the pressure < 15 atm, since adsorptive sites are occupied by water molecule. As compared with the CO_2_ sorption curve in water ([Supplementary-material supplementary-material-1]), the slight CO_2_ uptake is mainly attributed to the CO_2_ adsorption on the carbon surface. Obviously, the carbon material with different water ratios presents similar CO_2_ uptake before 15 atm, and the slight difference should be due to the dissolved CO_2_. It was interestingly found that a small step occurred under 15.1 atm and a large step happened under pressure > 25 atm. The first step around 14-15 atm observed on all wet CMK-3 should be assigned to the gas hydrate formation. The uptake at the first step increases dependent on the* Rw* value obviously. When* Rw *> 1, water would overflow the pore volume and occupy the gap of the particles to form the gas hydrates under relatively lower pressure comparing with the formation in the confined space. Based on the equation *dG* = −*SdT* + *VdP* + *λdA*, the smaller the gas hydrate crystal form, the higher the excess surface Gibbs free energy. According to the equation (∂*G*/∂*p*)_*T*_ = *V*, a higher formation pressure is needed when small crystal forms. Similar gas hydrate formation pressure on the porous material to that in bulky water system implies that the gas hydrates are formed out of the pore.

The second step that occurred at relatively higher pressure should be attributed to the gas hydrate formation. However, it was noted that the second step had a similar uptake increase no matter what the* Rw* value was. The molar ratio of water to CO_2_ defined as *x* was listed in Table 1. It is obvious that the *x* value (7.67) is higher than the theoretical one (5.75) when water is completely converted into the CO_2_ hydrate. The reason is that 5^12^ small cages are preferably occupied by water molecule, and only 6 large cages are retained for CO_2_ in a unit cell. In past, this excess uptake was attributed to (1) excess adsorption in the gap between the gas hydrate and carbon surface or (2) the formation of incomplete cages. In the present case, we propose more feasible mechanism that the excess uptake comes from the CO_2_ condensation in the mesopore of CMK-3, where water is replaced by CO_2_. This mechanism is also supported by other experimental results. When* Rw *= 2.01 (Figure 2(e)), water outside the pore is firstly converted to the CO_2_ hydrate under relatively lower pressure of 14 atm at 273 K. With pressurizing CO_2_, fragments of water clusters start to form in the pore in order to reduce the surface Gibbs free energy. These fragments and microcrystals tend to combine and grow up out of the pore. However, no sharp increase of uptake could be found under this condition, indicating that the migration of gas hydrate fragments cannot occur independently, unless accompanied with the CO_2_ condensation in the mesopore. Cooperating with the CO_2_ condensation, the water cluster migrates from inside pore to outside for further growth of the CO_2_ hydrate crystals. Meanwhile, the pore gradually becomes empty and allows for the CO_2_ condensation to occur. By comparing the CO_2_ uptake under the two steps and the CO_2_ adsorption amount on dry carbon (Table 1), it is clear that the mechanism we proposed here is reasonable. When subtracting the adsorptive amount of CO_2_ from the total uptake, the gas hydrates obtained on all wet CMK-3 show reasonable *x* value to the theoretical one. The result could also be obtained from the desorption branch of isotherm curves. As shown in [Supplementary-material supplementary-material-1], the desorption branch of CO_2_ on CMK-3 (*Rw* = 0.96) shows an obvious hysteresis loop, which is much different from the desorption branch of CO_2_ on dry CMK-3. To make it clearer, the estimated gas hydrate amount formed on CMK-3 (6.83 mmol/g) was deducted from the desorption branch. It was found that, when the pressure is >23.7 atm, the desorption branch meets well with that on dry CMK-3, indicating a similar desorption mechanism. When the pressure is decreased, the CO_2_ uptake on wet CMK-3 decreases more quickly than that on dry sample. During this step, the gas hydrate decomposes into free water molecule to reoccupy the pore of CMK-3 and accelerate the CO_2_ desorption. When calculating the CO_2_ uptake by desorption on dry carbon between 12.1-23.7 atm, the released gas amount by gas hydrate decomposition is about 6.91, which is consistent with the value estimated from the adsorption branch.

When similar experiments were carried out on AC with irregular slit pore size around 2 nm, clearly different isotherm curves were obtained. The CO_2_ isotherm (Figure 2(f)) on dry AC showed type I curve because of its small pore size (2 nm). Different from the isotherm on wet CMK-3, the CO_2_ isotherm on wet AC showed only one inflexion. When the water loading amount is equal to the pore volume, the adsorptive amount exhibits a smooth increase around 15 atm at 273 K, which is very different from the sharp increase at 14 atm observed on wet CMK-3. It is notable that the uptake on wet AC with* Rw* = 1.58 is similar to that on its dry sample. It was deduced that the smooth increase is attributed to the replacement of water by CO_2_. When* Rw* = 1.58, the uptake reaches the maximum value on dry AC, indicating that no water is converted to gas hydrates. The same result was observed on AC with* Rw* = 2.28. In this case, although the water loading obviously exceeded the pore volume of AC, no gas hydrate formation was observed around 14 atm, which was different from the results on CMK-3. The water out of the AC pore cannot form gas hydrates even after 48 h. The reason may be attributed to special porous structure of AC. The pore in AC has a V shape. The water replaced by CO_2_ could be located on the top of the V pore, where the pressure for the gas hydrate formation is too high to reach when the CO_2_ condensation takes place at the bottom of the pore.

A small hysteresis loop was observed in the desorption branch (*Rw* = 2.28) ([Supplementary-material supplementary-material-1]), which is very different from the CO_2_ desorption branch on dry AC, indicating a different desorption mechanism. In this case, the adsorbed CO_2_ molecule is easier to depart than that on dry carbon. At 1 atm, the CO_2_ desorption could achieve more than 99%. When the pressure decreases, the adsorbed CO_2_ dissociates from the surface and is replaced by water molecule. Under the replacement effect of water, the CO_2_ dispersion occurs more easily than that on dry AC, attributing to both enthalpy and entropy. On one hand, rebinding of water to carbon surface compensates the enthalpy penalty when CO_2_ disperses from the surface. One the other hand, the increase of translation entropy caused by CO_2_ desorption is higher than the reduction of translation entropy caused by H_2_O adsorption, driving the process proceeding forward. When* Rw* increases to 3.1, partial water out of the V pore could form gas hydrates at relatively lower pressure, driving water in the pore to participate in the CO_2_ hydrate formation. The isotherm curve (*Rw* = 3.1) shows that the sharp increase which occurred at about 15 atm corresponds to the CO_2_ hydrate formation. By calculating molar ratio of water to gas, the value of* x *= 7.65 is well consistent with the theoretical one (7.67), indicating that water on AC is completely converted to the gas hydrate under this condition. It was also noticed that, after subtracting the uptake caused by the gas hydrate formation, the isotherm curve (*Rw* = 3.1) is similar to others (*Rw* = 1.58 and 2.28), suggesting that the mechanism of CO_2_ adsorption on AC with pressure > 15 atm under different* Rw* is similar. The desorption branch (*Rw *= 3.1) shows a small hysteresis loop ([Supplementary-material supplementary-material-1]). The sharp decrease of CO_2_ uptake occurs at about 13.9 atm corresponding to the hydrate crystal dissociation and CO_2_ desorption from the nanopore accompanied with the reoccupation of water molecule in the nanopore. Similar to the case of CMK-3, the CO_2_ dispersion process is promoted by the replacement of water molecule after the gas hydrate decomposition.

In order to distinguish the mechanisms corresponding to the gas uptake obtained from thermodynamically static studies in each step, kinetic adsorptive experiments were carried out. Both of the gas adsorption and hydrate formation are exothermic processes. The gas hydrate formation process is much slower than the adsorption process, because the former includes the inducing period for the nucleation and long period for the crystal growth. Therefore, the inducing period and slow growth rate with heat release are unique features in the hydrate formation. Kinetic curves with temperature changes are shown in Figures 2(g) and 2(h). From the kinetic curve, it was found that temperature peaks which occurred on both materials are accompanied with inflection points of uptake. As compared with blank experiment ([Supplementary-material supplementary-material-1]), when loading CO_2_ on dry CMK-3, only one temperature peak around 18°C was observed, attributing to the release of adsorptive heat, gas expanding heat, and heat transferred from the environment. In the case of* Rw* = 0.96 and 2.01, the first peak of temperature decreases to about 9°C because of high capacity of water. On wet CMK-3, the second inflection point occurs after about 20 min, and slow decline should be attributed to the phase transferring heat.

A similar trend of temperature changes was observed on AC as well. When loading CO_2_ on dry AC, the temperature peak reaches about 45°C, which is much higher than that on CMK-3 due to higher adsorptive amount and stronger potential intensity of the pore in AC. When loading CO_2_ on wet AC, the first temperature peak decreases to 20°C, because of high heat capacity of water. When* Rw* = 1.58, only one temperature peak corresponding to a sharp uptake increase without inducing time was observed. This is strong evidence for the conclusion that CO_2_ hydrates cannot be formed in this case. When* Rw* = 3.1, the peak of temperature around 8°C occurs with relatively longer platform, indicating that the process is accompanied with a slow exothermic process assigned to the gas hydrate formation.

In order to obtain the accurate value of the heat, the isotherm curves (Figures [Supplementary-material supplementary-material-1]-[Supplementary-material supplementary-material-1]) at different temperatures were collected for phase changing enthalpy analysis. According to the Clausius-Clapeyron equation, adsorptive enthalpy on CMK-3 with different* Rw* was obtained (see Supporting Information for more details). The enthalpy of the first step assigned to the hydrate formation out of pore is 64.7 kJ/mol. The enthalpy of the second step is 31.0 kJ/mol, which is lower than the enthalpy (64.7 kJ/mol) of the CO_2_ hydrate formation, but higher than CO_2_ condensation enthalpy (19.8 kJ/mol) derived from the CO_2_ adsorption on dry CMK-3. Since the enthalpy is not related to the pathway, the enthalpy of the entire process could be calculated from the initial state (gases CO_2_ + liquid water) and the final state (condensed CO_2_ + CO_2_ hydrate). The total calculated enthalpy of the second step is 32.0 kJ/mol, which is amazingly consistent with the value calculated according to the Clausius-Clapeyron equation (31.0-32.6 kJ/mol). These results further support our conclusion that the condensation and hydrate formation occur simultaneously at the second step. Similarly, by calculating the enthalpy from isotherm curves on wet AC (*Rw* = 3.1) at different temperatures ([Supplementary-material supplementary-material-1]), the enthalpy of the phase transfer corresponding to the sharp increase of uptake is 68.0 kJ/mol. Similar enthalpy on wet AC (20.0 kJ/mol,* Rw* = 1.58) and on dry AC (23.0 kJ/mol) indicates the same adsorptive mechanism.

It is worth noting that the initial of uptake (8.2 mmol/g) on wet CMK-3 agrees with the adsorptive amount (9 mmol/g) estimated from surface area of CMK-3 (998 m^2^/g), but it is obviously lower than that on dry sample (21 mmol/g). When introducing CO_2_ in the system, the first process which happened is that CO_2_ replaces part of water adsorbed on the material surface. After the first process, CO_2_ molecule adsorbs on the surface of the pore, and the center of the pore is still filled by water molecule. Under experimental pressures with inducing time, the water in the pore is supersaturated by hydrate nucleus. Thus, CO_2_ molecule prefers to condense in the center of the pore, and the hydrate nucleus tends to grow up to large crystals. Under these conditions, water in the pore continuously transfers out for the conversion, and CO_2_ is continuously condensed in free space. The kinetic curves of wet CMK-3 indicate that the surface adsorption by CO_2_ occurs first, and then the gas hydrates are formed along with the CO_2_ condensation. The final result is that water in the pore is replaced by condensed CO_2_.

With regard to AC, it is different from CMK-3. The initial adsorptive amount on wet AC (*Rw* = 1.58 and 3.1) is similar to that on dry AC, indicating a complete replacement at first. On CMK-3, initially CO_2_ could only replace the water adsorbed on the carbon surface, while, on AC, CO_2_ could replace almost all water in the pore. This is because the pore size of these two materials is different. The pore size of AC (2 nm) is smaller than that of CMK-3 (3.8 nm), resulting in stronger potential intensity in the pore of AC and leading to the CO_2_ condensation in the pore of AC. As aforementioned, no gas hydrate could be formed on wet AC (*Rw* = 1.58). By increasing water loading amount, it was found that gas hydrates are formed and approach the theoretic hydrate volume. It is understandable that, upon increasing* Rw*, water is replaced and squeezed out of wedgy pore by CO_2_ molecule. Part of water out of the pore could form CO_2_ hydrates under this condition. It is very interesting that, once the gas hydrates start to form out of the pore, water inside the pore is driven out of the pore to participate in the CO_2_ hydrate formation. Comparing with the CO_2_ adsorption on CMK-3 having a dependent replacing process with the gas hydrate formation, the replacement on AC could occur anytime, i.e., before, during, or after the gas hydrate formation.

After initial replacement process on CMK-3 and AC, whether water in middle layer of pores is replaced by CO_2_ with pressurizing CO_2_ is determined by the pore size. In small pores, after initial adsorption of CO_2_ on the carbon surface, there is no space for water molecule, and water is squeezed out of the pore completely. When the pore size is large enough for water to occupy middle layer of the pore, the binding energy of H_2_O-H_2_O and H_2_O-CO_2_ is 25 kJ/mol and 19.8 kJ/mol, respectively, calculated by DFT. Obviously, the CO_2_-CO_2_ interaction (8.3 kJ/mol) is weaker than that of CO_2_-H_2_O or H_2_O-H_2_O, meaning that the CO_2_ insertion is not a spontaneous process. To overcome the enthalpy barrier, the CO_2_ potential in the gas phase must be enhanced by increasing the CO_2_ pressure. When the replacement of water by CO_2_ in middle layer of the pore is achieved, CO_2_ is located in the pore and condensed. The condensation pressure in 2 nm pore is obviously lower than that in 3 nm pore, since the overlap potential is generated by carbon walls. As observed from static adsorption experiments, the pressure for the water replacement by CO_2_ in 2 nm slit pore is about 16 atm. In contrast, in 3.8 nm pore of CMK-3, no replacement could be observed. The pressure inducing this process in 3.8 nm pore is much higher than that for the gas hydrate formation. Therefore, the gas hydrate formation occurs firstly, leading to the transportation of water molecule from inside pore to outside in order to participate in the formation of CO_2_ hydrate crystals.

**Figure 3:**
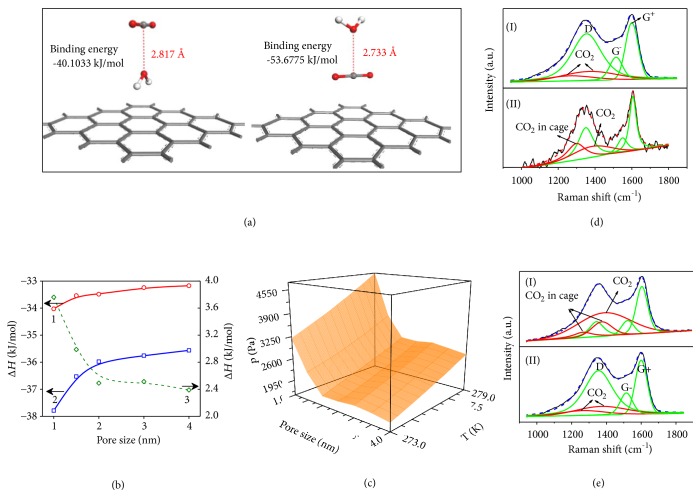
*Simulation and calculation studies of gas hydrate formation process and in situ Raman studies*. (a) Comparison of CO_2_-H_2_O adsorptive structure on graph (001). (b) Adsorption enthalpy change of water and CO_2_ on slit pore model with different pore size: (1) for CO_2_; (2) for water; and (3) is the difference of enthalpy between water adsorption and CO_2_ adsorption in slit pore with different pore size. (c) Relationship between pressure, pore size, and temperature when the Gibbs energy of the process is less than zero. (d)* In situ* Raman spectra after (I) loading CO_2_ on CMK-3 (*Rw *= 1) immediately and (II) loading CO_2_ on CMK-3 (*Rw*=1) for 8 h. (e)* In situ* Raman spectra after loading CO_2_ on (I) AC-carbon (*Rw *= 3.1) for 8 h and (II) AC-carbon (*Rw *= 1.58) for 8 h.

Based on static and kinetic studies, the process of introducing CO_2_ in wet carbon systems includes an initial replacement of water by CO_2_ on the first layer of carbon surface, the replacement of water in middle layer of the pore, and the formation of CO_2_ hydrates. Then, we calculated the entropy, enthalpy, and free energy extracted from ab initio calculations of CO_2_-water system confined in layered graphene model with slit size of 1, 1.5, 2, 3, and 4 nm (Figure 3(a)). The replacing process could occur at the carbon surface before the gas hydrate formation, although the carbon atom on the surface of CMK-3 has higher binding energy with H_2_O than with CO_2_ as shown in Figure 3(b). Therefore, the replacement process of water by CO_2_ should be dominated by the entropy.

The entropy of free molecules (CO_2_ and H_2_O) and adsorbed molecules on the carbon surface and in CO_2_ hydrates was calculated from their vibrations (Figures [Supplementary-material supplementary-material-1] and [Supplementary-material supplementary-material-1]). The total entropy is a sum of translational, rotational, and internal vibrational components: *S*_tot_ = *S*_trans_ + *S*_rot_ + *S*_vib_. Six vibration modes of water and CO_2_ were obtained, corresponding to bending vibration, symmetric stretching vibration, asymmetric stretching vibration, and rotational vibration. The detailed frequency is listed in [Supplementary-material supplementary-material-1]. When comparing the frequency of molecules before and after the adsorption, it was found that obvious changes occur at low frequency region corresponding to rotational vibration. The water rotational vibration is much restricted in carbon pore especially micropore, which accounts for 50% loss of entropy. In contrast, the CO_2_ rotational frequency is not changed obviously, meaning that the entropy of water replacement by CO_2_ on the carbon surface is favorable, which could compensate the loss of enthalpy. By taking account of the pore size effect, the relationship between pressure, pore size, and temperature when the Gibbs energy of the process is less than zero is shown in Figure 3(c). Obviously, the larger the pore size and the higher the temperature, the higher the pressure needed to make Gibbs free energy less than zero. In addition to the entropy-driven mechanism for the replacement process, in large pore, the replacement of H_2_O by CO_2_ in single layer region could be spontaneously driven not only by entropy but also by enthalpy. As shown in Figure 3(a), when CO_2_ molecule is tiled on the carbon (001) mesh along the C-O-C orientation, the *π*-*π* stacking interaction enables one side of the *π* electrons to focus near the carbon surface, while the opposite side of *π* electrons is weakened, leading to the situation that lone pair electrons from the O_H2O_ atom have strong interaction with positively charged C_CO2_ atom. If lone pair electrons of water directly bind with the surface of carbon, H_H2O_ must direct to CO_2_ molecule. If lone pair electrons of water direct to C_CO2_, the *π* electrons of CO_2_ would still have strong interaction with the carbon surface. When compared with the other structure of C-H_2_O-CO_2_, simulation results show that the C-CO_2_-H_2_O structure is more stable. Thus, the stable configuration makes the replacement of water by CO_2_ feasible.

To further confirm the conclusion that CO_2_ hydrates could be formed outside the pore under specific conditions,* in situ* Raman studies were performed on CMK-3 and AC. The Raman spectrum of these materials displays characteristic peaks in the spectral range of 1200 to 1800 cm^−1^. The G feature has a characteristic peak around 1582 cm^−1^, assigning to all carbon structures having* sp*^2^ hybridization [26]. The band around 1351 cm^−1^ (*λ* = 532 nm) is known as the D band (defect-induced), and it requires a structural defect to be active in honeycomb carbon lattice. On account of the curvature in contrast to the perfect honeycomb lattice of graphite, the G band splits into the G+ and G− bands centered around 1571 and 1593 cm^−1^, respectively (Figures 3(d) and 3(e)). Then, the* in situ* Raman spectra were collected on CMK-3 with water ratio of 1 (*Rw *= 1). At the initial loading of CO_2_, the CO_2_ adsorption occurs quickly according to the kinetic study results, and the Raman spectrum is separated into several parts (Figure 3(d)(I)). The Raman spectrum of CO_2_ in adsorptive phase shows symmetric stretching (1286 cm^−1^) and bending vibration (1389 cm^−1^). When CO_2_ molecule is enclosed by the hydrate network, the spectrum is broadened and red shifted (Figure 3(d)(II)). When the hydration is completed, symmetric stretching and bending vibration peaks of CO_2_ at 1278 and 1386 cm^−1^ shift to 1277 and 1382 cm^−1^, respectively, assigning to CO_2_ vibration in 5^12^ cages. Similar changes were observed on AC as well. Symmetric stretching and bending vibration peaks (Figure 3(e)(I)) shift when water loading ratio is 3.1, indicating the CO_2_ hydrate formation. At* Rw *= 1.58, no obvious shifts of CO_2_ vibration peaks (Figure 3(e)(II)) were observed, indicting no hydrate formation under this loading ratio, which agrees well with earlier experimental results.

Based on our experimental results on both representatives (CMK-3 and active carbon AC) and DFT studies, the mechanism of gas hydrate formation on porous materials has been well revealed. The water coming from the inner pore could occupy the gap between particles, and the formed gas hydrate crystals fill the empty space. The established conclusion not only provides a new theory to the gas hydrate formation on porous materials, but also offers a reference for further application of gas separation.

**Figure 4:**
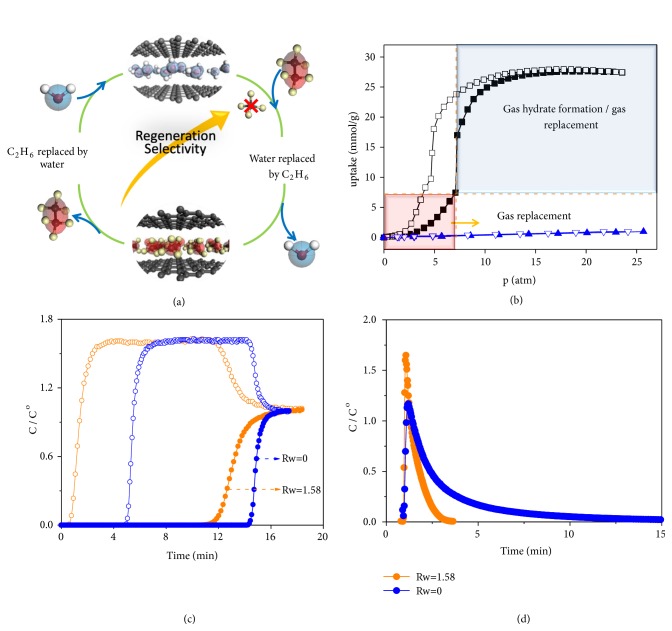
*Experimental studies of CH*_*4*_ */C*_*2*_ *H*_*6*_ *separation*. (a) Scheme of replacement during the gas adsorption process. (b) Gas isotherm curves on AC at 273 K with* Rw* = 1.58. Blue curve and black curve represent methane and ethane, respectively. The filled symbols represent adsorption branch, and the blank symbols represent desorption branch. (c) Breakthrough curve of CH_4_/C_2_H_6_ mixture on AC at 273 K under 1.5 MPa with a flow rate of 100 mlL/min. (d) Regeneration curves under atmosphere pressure when purging with N_2_ at 273 K with a flow rate of 100 mL/min. In (c) and (d), the filled symbols represent C_2_H_6_, and the blank symbols represent CH_4_.

The water molecule preadsorbed in the nanopore plays the role of recognizer to different gas (Figure 4(a)). After investigating several gas adsorption/desorption isotherms (CO_2_, CH_4_, and C_2_H_6_ in Figures [Supplementary-material supplementary-material-1]-[Supplementary-material supplementary-material-1]) on wet carbon materials, it was found that the replacing pressure is related to the gas type. In the case of CH_4_, the water in the pore could only be driven out to form gas hydrate crystals out of pore at high pressure. In comparison to CH_4_ and C_2_H_6_ (Figure 4(b)) on AC (*Rw* = 1.58), C_2_H_6_ could replace water adsorbed in the pore at a relatively low pressure. By utilizing this difference, the enhancement of CH_4_/C_2_H_6_ selectivity from 4 (on dry AC) to 24 (on wet AC) was achieved by calculating from the breakthrough curves (Figure 4(c)). Only C_2_H_6_ could replace the water adsorbed on AC at current experiment conditions on account of its high binding energy and low entropy penalty. Due to the low binding energy in the pore, CH_4_ cannot simply replace water, leading to an ultralow adsorptive amount on wet AC in comparison with that on dry AC ([Supplementary-material supplementary-material-1]). It should be noted that our finding here is much different from the gas separation based on gas hydrates. The enhancement of selectivity could only be achieved in the case that gas hydrates do not form. Once gas hydrates form, water would be driven out of pore to form the gas hydrate cluster, which increases methane competitive occupation in the empty pore of AC and the cage of formed gas hydrates. The increased methane uptake would reduce the CH_4_/C_2_H_6_ selectivity. Therefore, the enhanced gas separation in our study follows a different mechanism. More importantly, the regeneration time of C_2_H_6_ on wet AC reduces to 1/6 on dry AC (Figure 4(d)). Thus, by simply using water in the pore, the drawback of regenerating strong adsorptive gas on porous adsorbents that hinders their industrial applications was overcome.

When the C_2_H_6_ partial pressure decreases, the water molecule could reoccupy the pore to promote desorption of C_2_H_6_ molecule. This enhancement could also be understood from the entropy change of the whole process. When water molecule is replaced by gas molecule, the entropy penalty should be compensated by the enthalpy. The CH_4_ binding energy (-27.39 kJ) is much lower than that of C_2_H_6_ (-39.09 kJ) in the pore, so that water could only be replaced by C_2_H_6_. During the regeneration process, both of entropy and enthalpy are beneficial to the C_2_H_6_ desorption and water reoccupation in the pore of AC.

## 3. Discussion

Based on the thermodynamic and kinetic study on CO_2_ adsorption combined with DFT calculations, we have proposed a new mechanism of the gas hydrate formation on porous materials, which is quite different to previous hypothesis. We have discovered that the gas hydrates could not form and stay in the nanopore, and the excess gas observed in artificial gas hydrates resulted from the gas adsorption and condensation in the nanospace when water is driven out to form gas hydrates out of the pore. The established conclusion not only provides a theory to the gas hydrate formation on porous materials, but also offers a guideline for further application of gas hydrates in the gas separation. The significant improvement of gas selectivity and regeneration could be achieved by controlling the gas hydrate formation process accurately.

According to the new understanding, the method to enhance the gas hydrate formation by using hydrophobic porous materials should be reestimated. Upon the gas hydrate formation on porous materials, the preloaded water could be replaced by adsorbates such as CO_2_, methane, and ethane. Low packing density is always an issue limiting the application of porous materials in the gas storage. Our research by combining the gas storage in the pore and the gas hydrate formation in the empty space overcomes the bottleneck of low packing density and would greatly enhance the gas storage capacity of porous materials under practical conditions. Thus, the present mechanism might be applicable to guide the separation of other binary gas systems.

## Figures and Tables

**Table 1:** CO_2_ uptake during the gas hydrate formation process and corresponding hydrate number.

Sample	*Rw*	Uptake					Molar ratio of water to gas		
		Total uptake	Adsorptive amount	GH_1st_	GH_2nd_	GH_tol_	x_tol_	x_1st_	x_2nd_
C1	0	21.1	21.1	-	-	0	-	-	-
C2	0.96	27.9	21.1	1.38	5.45	7.83	7.80	7.85	7.65
C3	2.01	35.6	21.1	8.72	6.05	14.77	7.64	7.23	7.79
C4	2.39	38.8	21.1	12.50	5.23	17.43	7.48	7.96	7.29
A1	0	37.1	34.0	0	0	0	-	-	-
A2	1.58	34.6	34.0	0	0	0	-	-	-
A3	2.28	35.0	34.0	-	-	-	-	-	-
A4	3.10	56.5	34.0	-	-	22.50	7.65	-	-

GH_1st_, GH_2nd_, and GH_tol_ correspond to the uptake that resulted from the gas hydrate formation in the first step, second step, and sum of the two steps, respectively. *x*_1st_, *x*_2nd_, and *x*_tol_ are the gas hydrate numbers corresponding to the formed gas hydrate in the first step, second step, and sum of the two steps, respectively.

## Data Availability

All data needed to evaluate the conclusions in the paper are present in the paper and the Supplementary Materials. Additional data related to this paper may be requested from the authors.

## Supplementary Materials

Supplementary Materials Method, enthalpy calculation, and thermodynamic calculation and DFT study. Figure S1: N_2_ adsorption/desorption isotherms on (a) CMK-3 and (b) AC at 77 K. Insets are DFT pore size distributions. Figure S2: water vapor adsorption/desorption isotherms on dry samples of (a) CMK-3 and (b) AC at 293 K. Figure S3: CO_2_ adsorption/desorption isotherms in bulk water at 273 K. Figure S4: comparison of CO_2_ adsorption/desorption isotherms on CMK-3 at 273 K. The red blank squares represent the desorption branch after deducting an estimated gas hydrate formation value (6.83 mmol/g) from the real desorption branch. Figure S5: comparison of CO_2_ adsorption/desorption isotherms on AC with different Rw at 273 K. Figure S6: blank experiment of CO_2_ adsorptive kinetic temperature curve. Figure S7: CO_2_ adsorptive isotherms on CMK-3 at different temperatures when* Rw *= 0. Figure S8: CO_2_ adsorptive isotherms on CMK-3 at different temperatures when* Rw *= 0.96. Figure S9: CO_2_ adsorptive isotherms on CMK-3 at different temperatures when* Rw *= 2. Figure S10: CO_2_ adsorptive isotherms on AC at different temperatures when* Rw *= 0. Figure S11: CO_2_ adsorptive isotherms on AC at different temperatures when* Rw *= 1.58. Figure S12: CO_2_ adsorptive isotherms on AC at different temperatures when* Rw *= 3.1. Figure S13: vibration modes of adsorbed water molecule and CO_2_ on graphene. Figure S14: optimized structures of CO_2_ hydrate crystals based on calculations. Red represents oxygen atoms in CO_2_, grey represents carbon atom, and blue and light green represent oxygen and hydrogen in water, respectively. Figure S15: C_2_H_6_ adsorption/desorption isotherms on AC at 273 K with different* Rw* values. The filled symbols represent adsorptive branch, and the blank symbols represent desorption branch. Figure S16: C_2_H_6_ adsorption/desorption isotherms on AC at different temperatures when* Rw *= 1.5. Figure S17: CH_4_ adsorption/desorption isotherms on AC at 273 K with different* Rw* values. The filled symbols represent adsorptive branch, and the blank symbols represent desorption branch. Figure S18: CH_4_ and C_2_H_6_ adsorption/desorption isotherms on dry AC at 273 K. Table S1: physical parameters of samples. Table S2: vibration frequency of free molecules and adsorbed molecules. See References [27–33].
