# Supplementary material for: Understanding the Pathway of Gas Hydrate Formation with Porous Materials for Enhanced Gas Separation
Source: Research (Wash D C). 2019 May 28;2019:3206024. doi: 10.34133/2019/3206024 (PMC6750046; doi:10.34133/2019/3206024)
Supplement: Supplementary Materials — Method, enthalpy calculation, and thermodynamic calculation and DFT study. Figure S1: N2 adsorption/desorption isotherms on (a) CMK-3 and (b) AC at 77 K. Insets are DFT pore size distributions. Figure S2: water vapor adsorption/desorption isotherms on dry samples of (a) CMK-3 and (b) AC at 293 K. Figure S3: CO2 adsorption/desorption isotherms in bulk water at 273 K. Figure S4: comparison of CO2 adsorption/desorption isotherms on CMK-3 at 273 K. The red blank squares represent the desorption branch after deducting an estimated gas hydrate formation value (6.83 mmol/g) from the real desorption branch. Figure S5: comparison of CO2 adsorption/desorption isotherms on AC with different Rw at 273 K. Figure S6: blank experiment of CO2 adsorptive kinetic temperature curve. Figure S7: CO2 adsorptive isotherms on CMK-3 at different temperatures when Rw = 0. Figure S8: CO2 adsorptive isotherms on CMK-3 at different temperatures when Rw = 0.96. Figure S9: CO2 adsorptive isotherms on CMK-3 at different temperatures when Rw = 2. Figure S10: CO2 adsorptive isotherms on AC at different temperatures when Rw = 0. Figure S11: CO2 adsorptive isotherms on AC at different temperatures when Rw = 1.58. Figure S12: CO2 adsorptive isotherms on AC at different temperatures when Rw = 3.1. Figure S13: vibration modes of adsorbed water molecule and CO2 on graphene. Figure S14: optimized structures of CO2 hydrate crystals based on calculations. Red represents oxygen atoms in CO2, grey represents carbon atom, and blue and light green represent oxygen and hydrogen in water, respectively. Figure S15: C2H6 adsorption/desorption isotherms on AC at 273 K with different Rw values. The filled symbols represent adsorptive branch, and the blank symbols represent desorption branch. Figure S16: C2H6 adsorption/desorption isotherms on AC at different temperatures when Rw = 1.5. Figure S17: CH4 adsorption/desorption isotherms on AC at 273 K with different Rw values. The filled symbols represent adsorptive branc [file 3206024.f1.pdf]

# Supplementary Materials

## Understanding the Pathway of Gas Hydrate Formation with Porous Materials for Enhanced Gas Separation

**Jia Liu[1,2], Yajuan Wei[1,3], Wei Meng[4], Pei-Zhou Li[1], Yanli Zhao[1,*], and Ruqiang Zou[4,*]**

[1]Division of Chemistry and Biological Chemistry, School of Physical and Mathematical Sciences, Nanyang Technological University, 21 Nanyang Link, Singapore 637371

[2]Department of Chemistry, School of Science, Tianjin University, Tianjin 300072, China

[3]School of Chemistry, Nankai University, Tianjin 300071, China

[4]Department of Materials Science and Engineering, College of Engineering, Peking University, Beijing 100871, China

*Correspondence should be addressed to Yanli Zhao; zhaoyanli@ntu.edu.sg and Ruqiang Zou; rzou@pku.edu.cn

**METHOD**

CMK-3 was synthesized according to previous reports [27,28]. The $N_2$ isotherm curves were collected at 77 K by ASIQ purchased from Quanta-chrome USA. Surface areas were calculated by BET method. Pore size distributions were obtained by DFT method. The transmission electron microscopy (TEM) images were collected by FEI Tecnai G2 F20. Water vapor sorption experiments were conducted by the same equipment for determining water loading amount. Powder XRD pattern was record on MiniFlex600 Rigaku. $CO_2$ (purity 99.998%) purchased from Oxygen Air Liquid Singapore was used in all experiments. Static and dynamic adsorption experiments were measured by high pressure adsorption equipment purchased from AMC USA. The breakthrough experiment was carried out on home-designed facility. The sample corresponding to the dry sample weight of 2 g was compacted in the steel fixed bed with 25 cm length and 1 cm inner diameter. The environment temperature was controlled by thermostatic water bath. The feed gas flow rate was controlled by mass flow controller with precision of 0.1 mL/min purchased from Bronkhorst High Tech. The outlet gas components were detected by mass spectrometer RGA 200 purchased from SRS, USA. During the breakthrough experiment, the mole ratio of mixture gas $C_2H_6/CH_4$ is 1:1. The flow rate of feed gas was 100 mL/min (STP). During the regeneration, $N_2$ was used as purging gas with

flow rate of 100 mL/min.

Wet samples were prepared by dropping deionized water slowly into massed dry samples with continuously stirring until the added water weight meets the requirement. 0.25g dry sample was used in thermodynamic test and wet sample preparation. 0.25g water was used in $CO_2$ sorption experiments at 273K.

## ENTHALPY CALCULTION

The phase change pressure could be obtained from isotherm curves at different temperatures. The enthalpy change of the state transition can be determined using the following Clausius–Clapeyron equation, where *f* is the fugacity, T is the temperature (K), and *R* is the gas constant (8.314 J $mol^{-1}$ $K^{-1}$).

$$\Delta H = -R\left[\frac{d\ln f}{d(1/T)}\right]_n$$

## THERMODYNAMIC CALCULATION AND DFT STUDY

To get thermodynamic behavior of water-$CO_2$ system under two-dimensional confined space, we built a 3 x 3 super cell of graphene with 1, 1.5, 2, 3 and 4 nm vacuum space to simulate the real pore of activated carbon and CMK-3. The interaction energies were computed using GGA pseudo potential method [29,30] with PBE density functional theory and dispersion correction (DFT-D3BJ) [31-33]. TNP basis set was selected during all simulations. More than one orientation of water molecule was considered. To lighten the calculation task, only one-layer graphite was built to mimic the inner wall, which is enough for us to discuss the experiment results. In all the simulations, the graphite atoms are fixed at their respective initial positions and represent an inert wall of carbon materials. The kinetic energy cut off was set to 400 eV. The maximum atom force was set as 0.05 eV for convergence criterion toward the structure optimization.

The purpose of calculations was to build a diagram that presents the relationship of pressure, temperature and pore size to Gibbs free energy. The process of water replacement can be described as follows:

$CO_2 + H_2O_{ads} \rightarrow H_2O + CO_{2ads}$

Using free gaseous $CO_2$, $H_2O$ and empty surface of carbon as references, the Gibbs free energy changes of interest can finally be represented as:

$$\Delta G(T,p) = G_{H2O} - G_{CO2ads} - \mu_{CO2}(T,p) + \mu_{H2Oads}(T,p)$$

$$\mu_i = Ho(T) - TSo(T) + k_B T \ln p / po$$

where $H$o is enthalpy, $S$ is entropy, $p$ is pressure, $k_B$ is Boltzmann constant, $T$ is temperature and $\mu$ is chemical potential.

The Gibbs free energies for relevant species were calculated with the expression:

$$\Delta G(T, p) \ = E_{DFT} + E_{ZPE} + E_{DF} + \int CpdT - TS$$

where $E_{DFT}$ is the DFT calculated electronic energy in CASTEP, $E_{ZPE}$ is the zero-point vibrational energy, $E_{DF}$ is correction of dispersion force, and ∫Cpd$T$-$TS$ is the correction of entropy contribution. Harmonic approximation was selected to treat the adsorbate, and $PV$ contributions were neglected.

The entropy $S$ can be given by:

$$S = Nk \ln q + NkT\left(\frac{\partial \ln q}{\partial T}\right)_V$$

$$q_t = Nk\left\{\ln\left[\left(\frac{2\pi mkT}{h^2}\right)^{3/2}\frac{V}{N}\right]+\frac{5}{2}\right\}$$

$$q_r = \frac{8p^2 IkT}{h^2\sigma}$$

$$q_v = \prod_{i=1}^{N} \frac{e^{h\nu_i/2kT}}{e^{h\nu_i/2kT} - e^{-h\nu_i/2kT}}$$

where $q$ is partition function that consists of translation ($q_t$), rotation ($q_r$), and vibration ($q_v$). $m$ is molecular weight, $I$ is the moment of inertia about, σ is symmetry number, and ν is vibration frequency. The vibration mode and frequency of free molecules ($H_2O$ and $CO_2$) and adsorbed molecules are shown in Figures S13 and S15 and listed in Table S2.

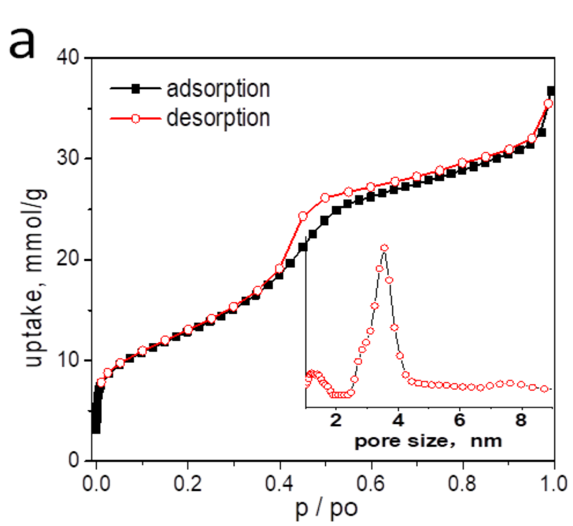


**Figure S1:** $N_2$ adsorption/desorption isotherms on (a) CMK-3 and (b) AC at 77K. Insets are DFT pore size distributions.

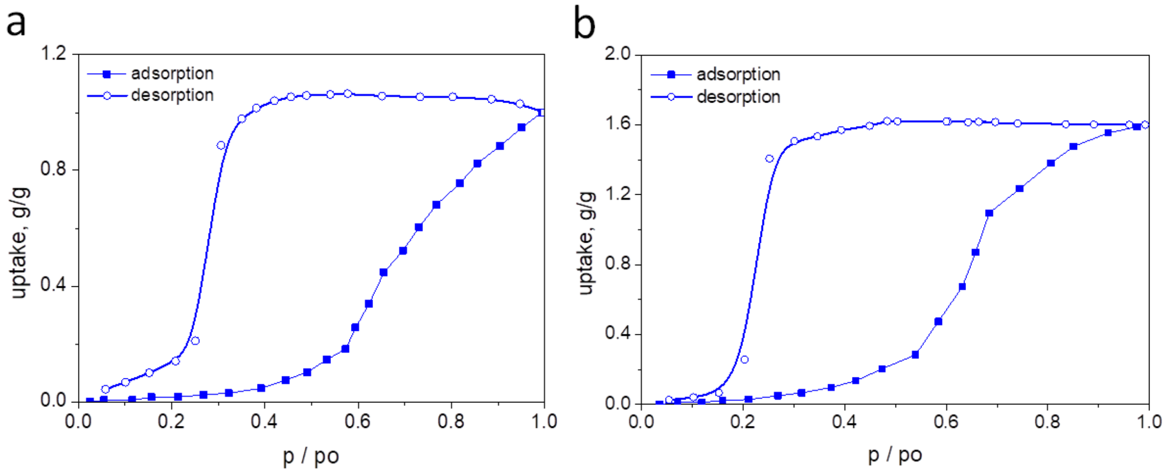


**Figure S2:** Water vapor adsorption/desorption isotherms on dry samples of (a) CMK-3 and (b) AC at 293K.

**Table S1:** Physical parameters of samples.

| Sample | Surface area, $m^2/g$ | Pore volume, $cm^3/g$ | Mean pore size, nm | Maximum water vapor uptake, $cm^3/g$ |
|---|---|---|---|---|
| AC | 3170 | 1.58 | 1-3 | 1.6 |
| CMK-3 | 998 | 1.08 | 3.8 | 1.0 |

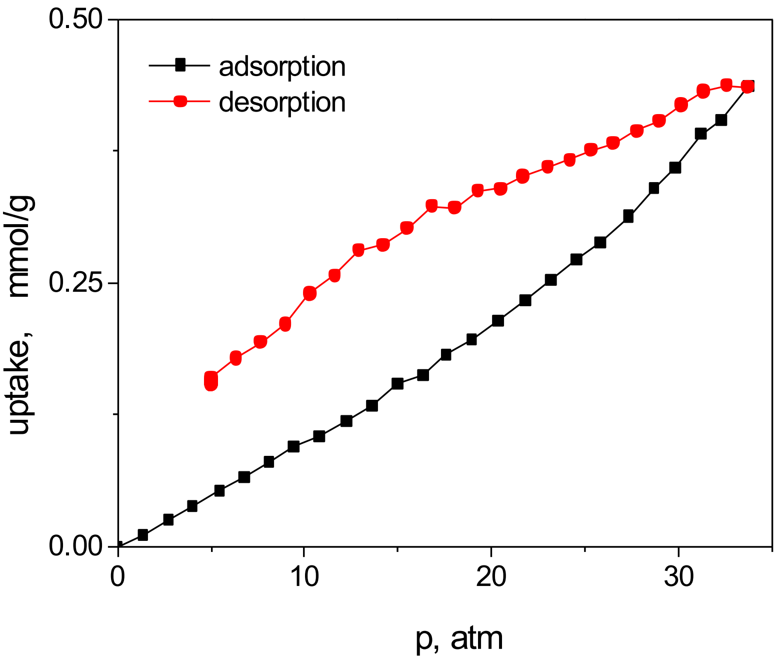


**Figure S3:** $CO_2$ adsorption/desorption isotherms in bulk water at 273 K.

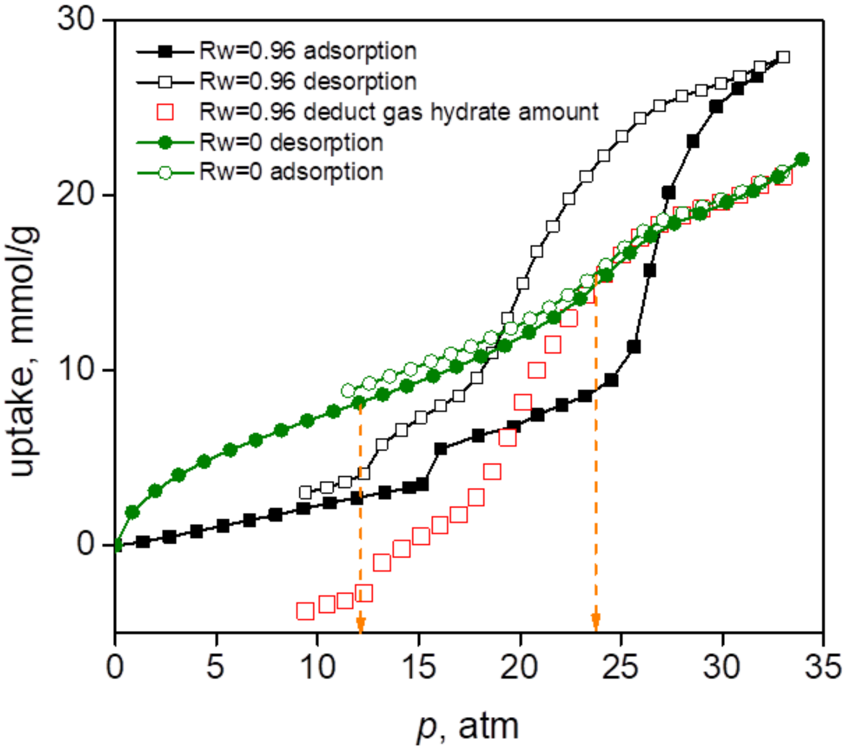


**Figure S4:** Comparison of $CO_2$ adsorption/desorption isotherms on CMK-3 at 273 K. The red blank squares represent the desorption branch after deducting an estimated gas hydrate formation value (6.83 mmol/g) from the real desorption branch.

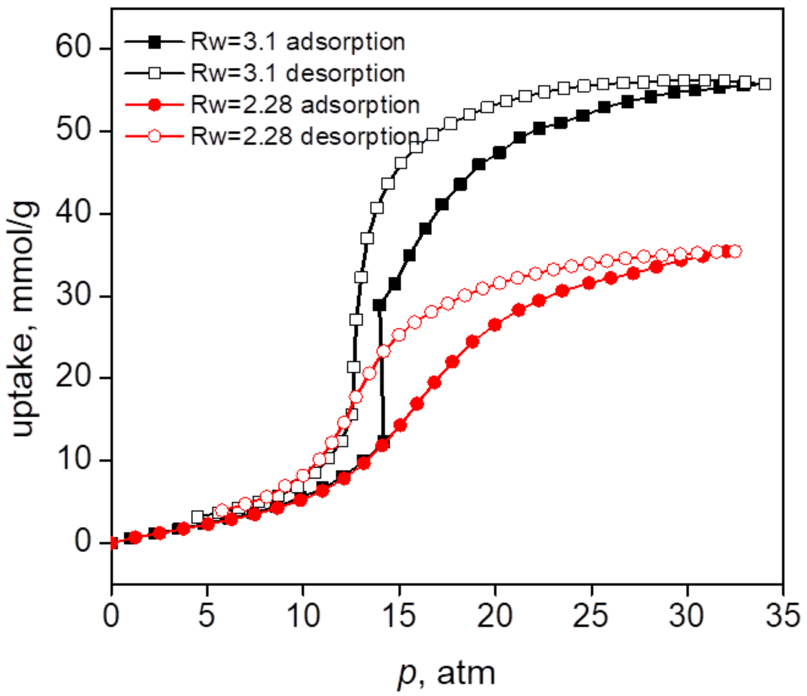


**Figure S5:** Comparison of $CO_2$ adsorption/desorption isotherms on AC with different Rw at 273 K.

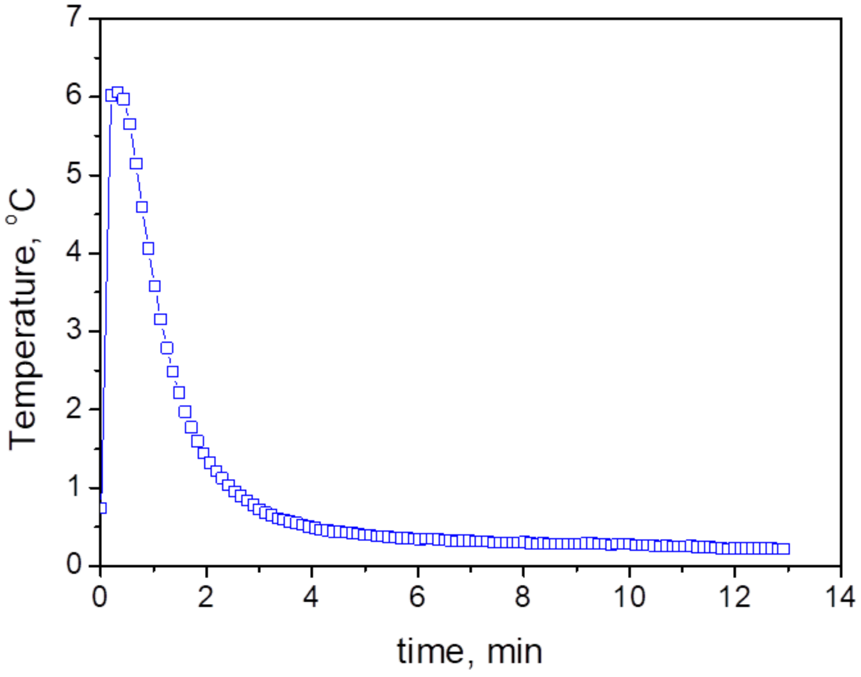


**Figure S6:** Blank experiment of $CO_2$ adsorptive kinetic temperature curve.

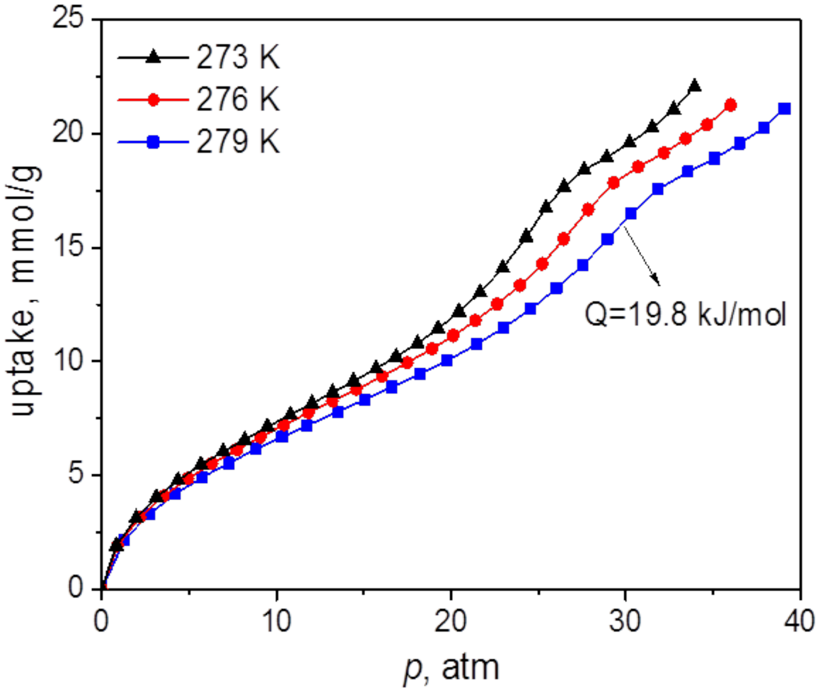


**Figure S7:** $CO_2$ adsorptive isotherms on CMK-3 at different temperatures when *Rw* = 0.

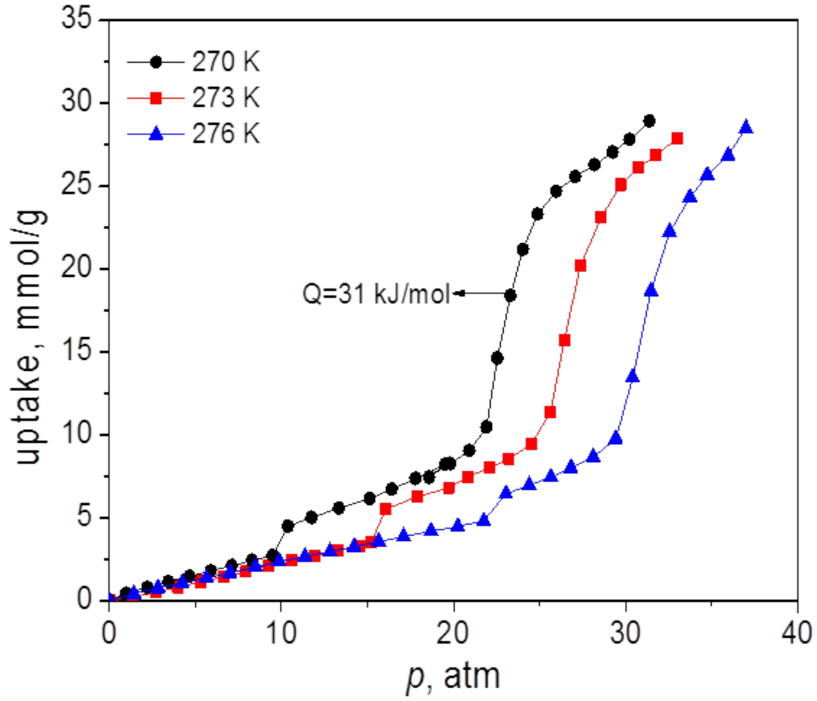


**Figure S8:** $CO_2$ adsorptive isotherms on CMK-3 at different temperatures when *Rw* = 0.96.

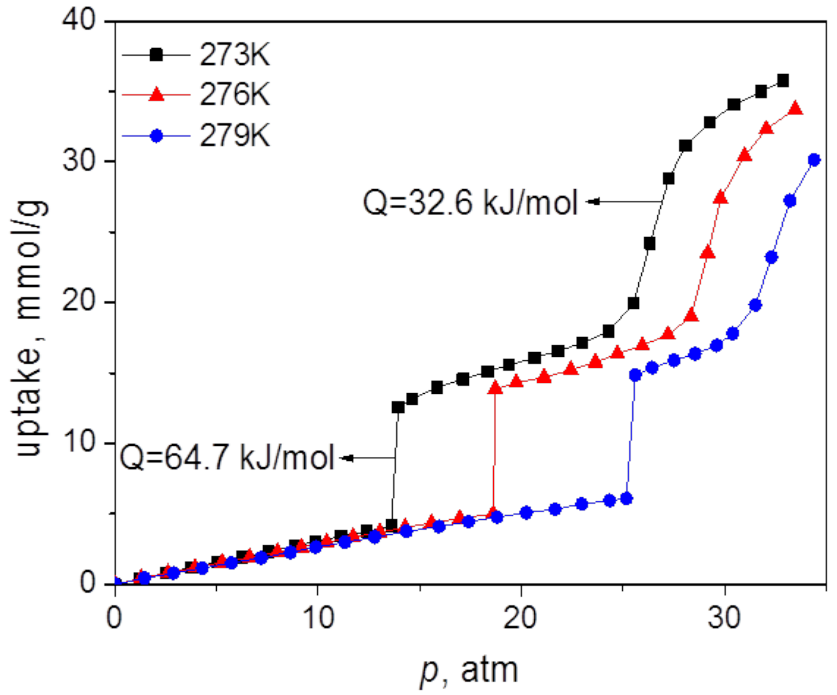


**Figure S9:** $CO_2$ adsorptive isotherms on CMK-3 at different temperatures when *Rw* = 2.

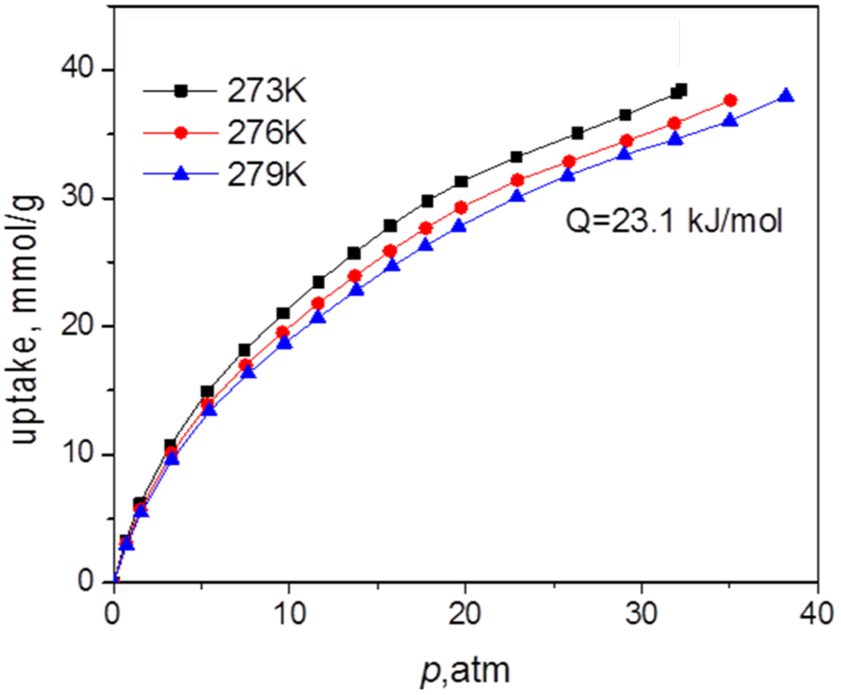


**Figure S10:** $CO_2$ adsorptive isotherms on AC at different temperatures when *Rw* = 0.

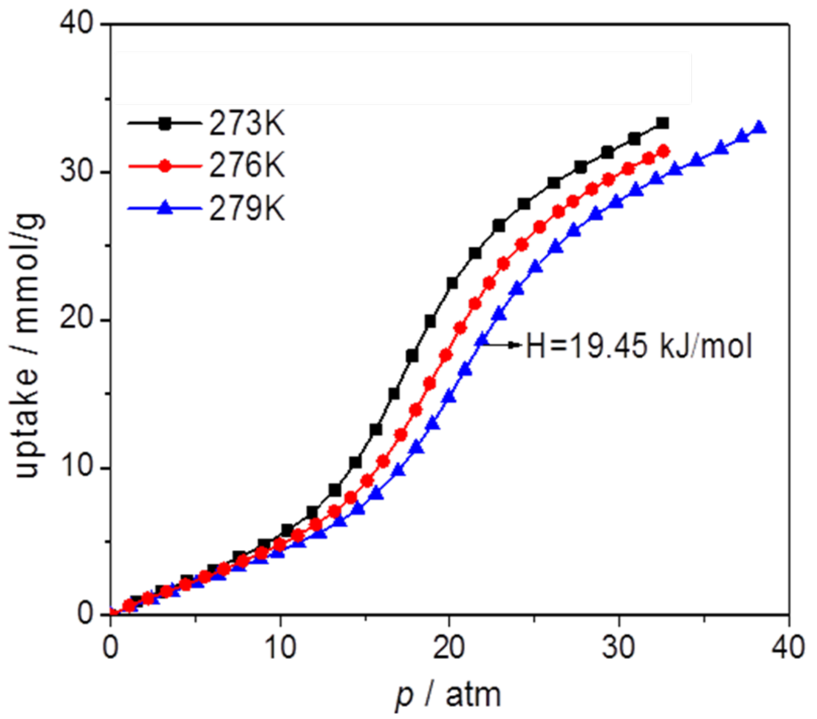


**Figure S11:** $CO_2$ adsorptive isotherms on AC at different temperatures when *Rw* = 1.58.

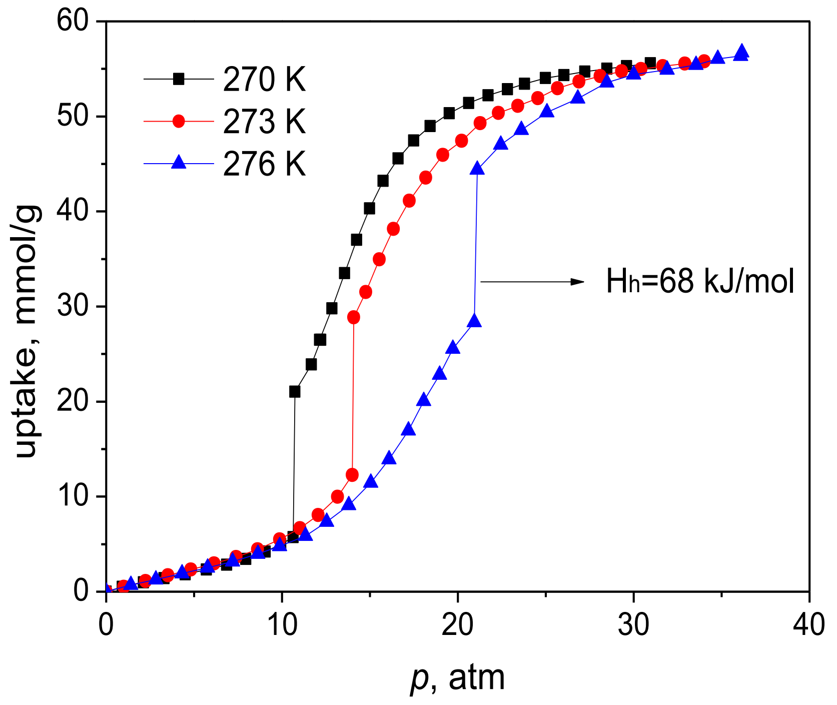


**Figure S12:** $CO_2$ adsorptive isotherms on AC at different temperatures when *Rw* = 3.1.

**Table S2:** Vibration frequency of free molecules and adsorbed molecules.

| species | Stretching vibration $cm^{-1}$ | | Bending vibration $cm^{-1}$ | | Rotation $cm^{-1}$ | |
|---|---|---|---|---|---|---|
| | symmetric | asymmetric | Out of plane | In plane | Out of plane | In plane |
| $CO_2$ | 1315 | 2348.8 | 657 | 657 | 16.89 | 16.89 |
| Adsorbed $CO_2$ | 1316 | 2342 | 636 | 639 | 61 | 90.03 |
| $CO_2$ in cage | 982 | $2106^S$ / $2193^L$ | $622^S$ / $635^L$ | $630^S$ / $640^L$ | 97 | 103 |

| species | Stretching vibration $cm^{-1}$ | | Bending vibration $cm^{-1}$ | Rotation $cm^{-1}$ | | |
|---|---|---|---|---|---|---|
| | **symmetric** | **asymmetric** | | **v1** | **v2** | **v3** |
| $H_2O$ | 3710 | 3816 | 1601 | 134 | 91 | 53 |
| Adsorbed $H_2O$ | 3674 | 3762 | 1653 | 392 | 397 | 444 |
| $H_2O$ of gas hydrate | 3780 | 3850-3900 | 1666 | - | 406 | - |

S: vibration of $CO_2$ in $5^{12}$ cage; L: vibration of $CO_2$ in $5^{12}6^2$ cage.

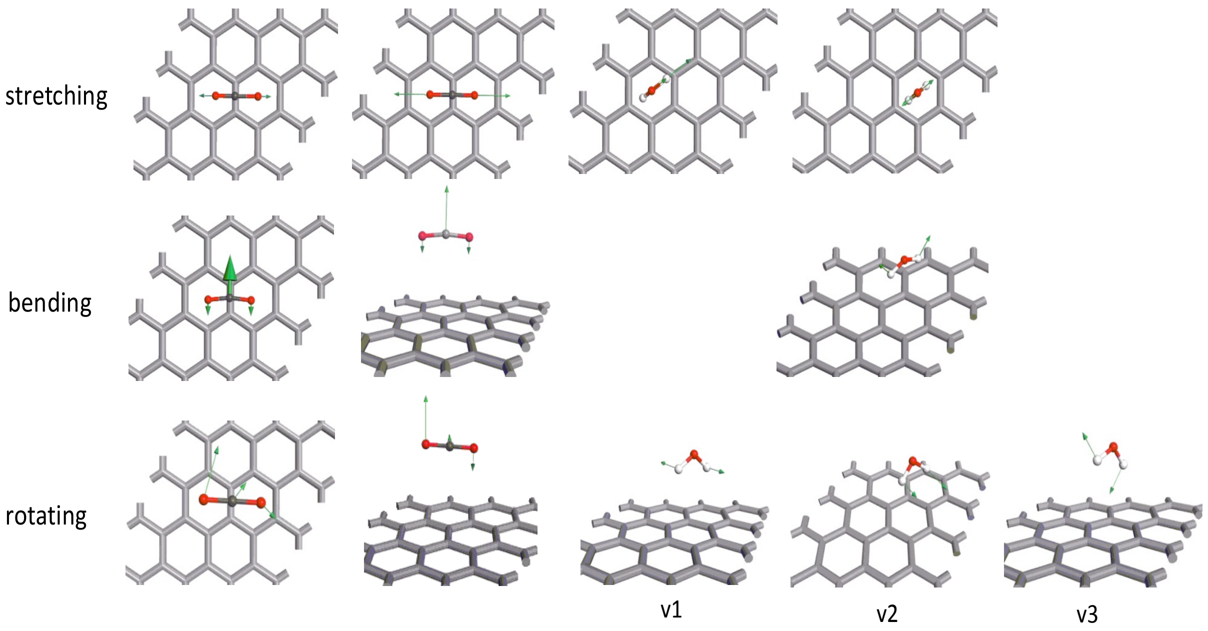


**Figure S13:** Vibration modes of adsorbed water molecule and $CO_2$ on graphene.

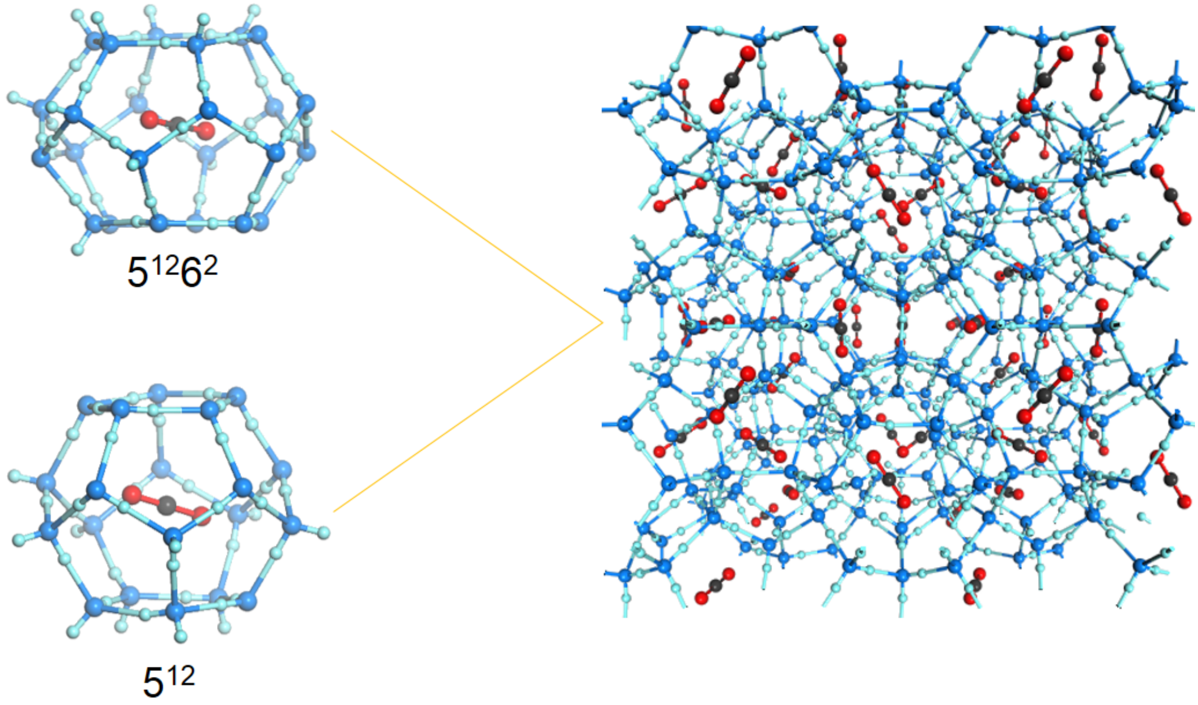


**Figure S14:** Optimized structures of $CO_2$ hydrate crystals based on calculations. Red represents oxygen atoms in $CO_2$, grey represents carbon atom, and blue and light green represent oxygen and hydrogen in water, respectively.

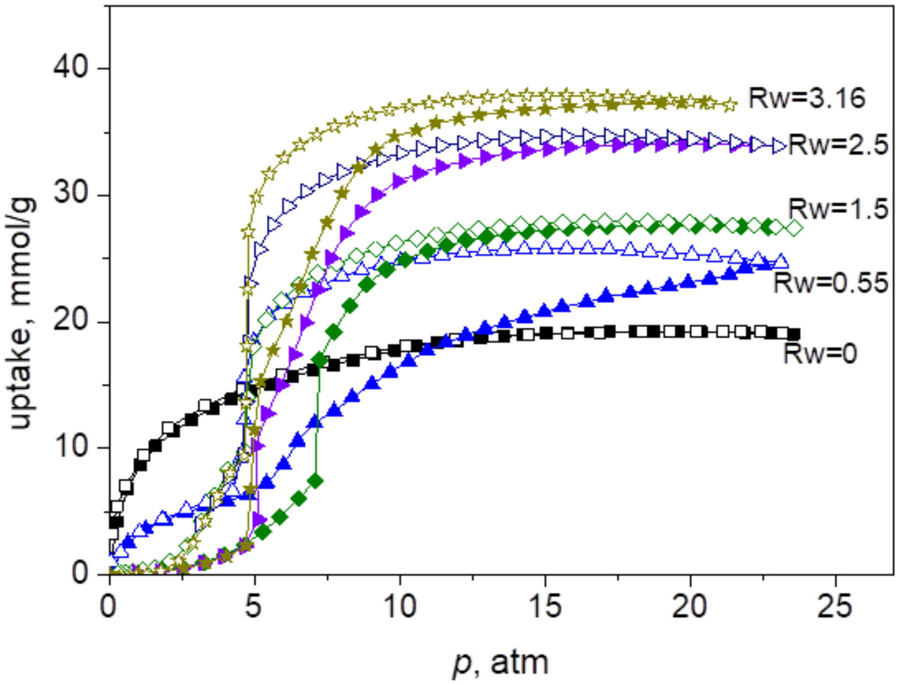


**Figure S15:** $C_2H_6$ adsorption/desorption isotherms on AC at 273K with different *Rw* values. The filled symbols represent adsorptive branch, and the blank symbols represent desorption branch.

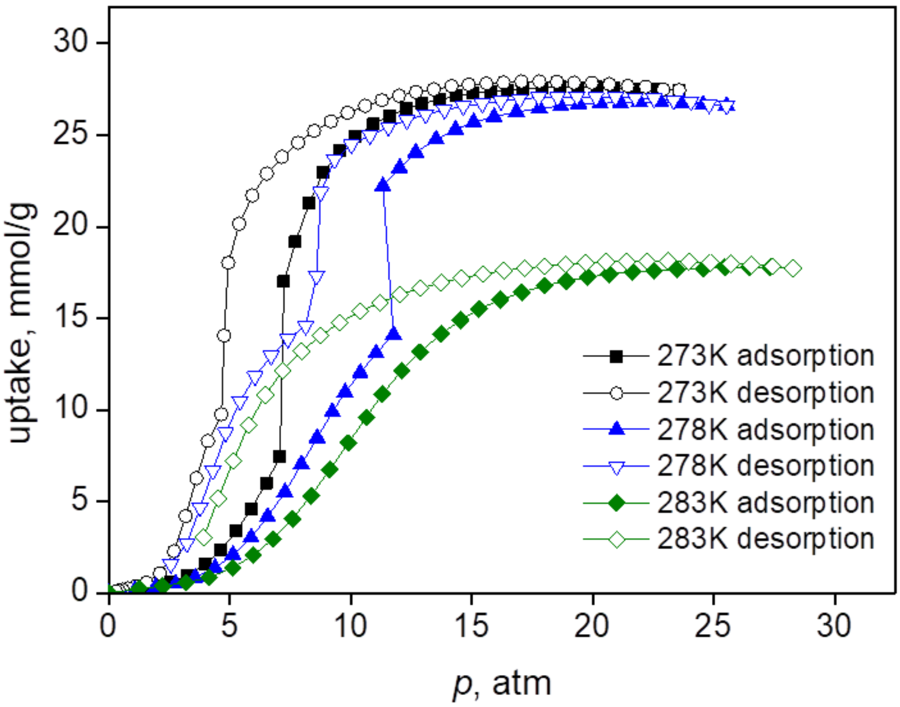


**Figure S16:** $C_2H_6$ adsorption/desorption isotherms on AC at different temperatures when *Rw* = 1.5.

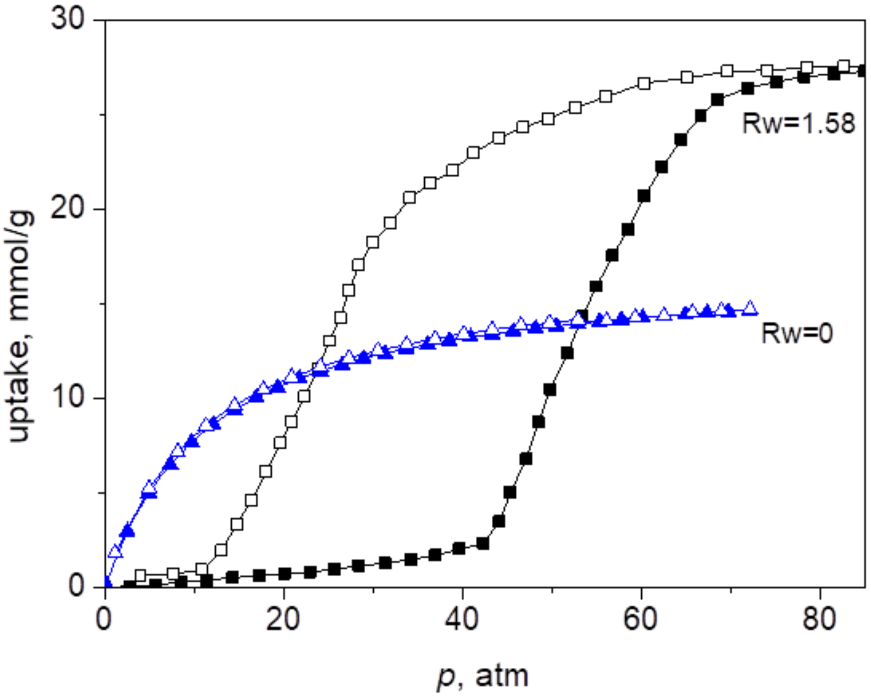


**Figure S17:** $CH_4$ adsorption/desorption isotherms on AC at 273K with different *Rw* values. The filled symbols represent adsorptive branch, and the blank symbols represent desorption branch.

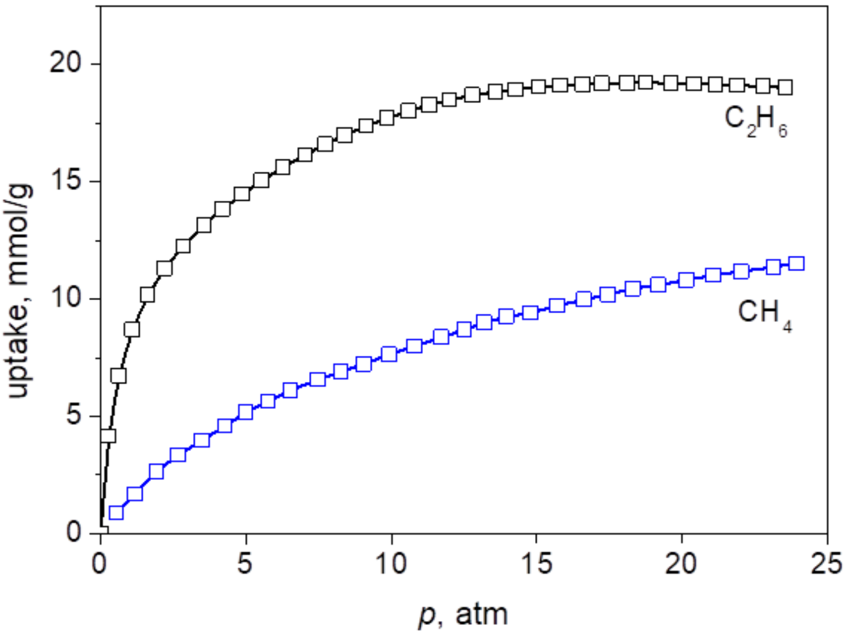


**Figure S18:** $CH_4$ and $C_2H_6$ adsorption/desorption isotherms on dry AC at 273 K.
